# Pituitary Adenoma With Apoplexy Presenting As Unilateral Third Nerve Palsy

**DOI:** 10.7759/cureus.40555

**Published:** 2023-06-17

**Authors:** Fatima Waqar, Ansharah Arif, Asmaa Muazzam, Areej Khan

**Affiliations:** 1 Internal Medicine, Abington Memorial Hospital, Abington, USA; 2 Internal Medicine/Family Medicine, California Institute of Behavioral Neurosciences & Psychology, Fairfield, USA; 3 Internal Medicine, Berkshire Medical Center, Pittsfield, USA

**Keywords:** pituitary apoplexy, pituitary tumor, unilateral ptosis, oculomotor nerve (cn iii) palsy, magnetic resonance imaging and pituitary adenoma

## Abstract

Pituitary adenomas are one of the most common intracranial tumors. Non-functioning macroadenomas are usually diagnosed when they cause symptoms due to the mass effect on surrounding structures. We present the case of a 48-year-old man who presented with a headache associated with ptosis of the right eye and right-sided blurry vision for three days. Initial computerized tomography (CT) scan of the head did not report a mass, acute infarct, or hemorrhage. He was given 325mg of aspirin for concern of a stroke while waiting for magnetic resonance imaging (MRI) of the brain, which was done the next day and revealed a pituitary macroadenoma with hemorrhage, mass effect, and compression of the optic chiasm consistent with pituitary apoplexy. He ultimately underwent trans-sphenoidal resection of the tumor; however, his surgery was delayed for five days as he had received a high dose of aspirin in the Emergency Room. His adrenocorticotropic hormone (ACTH), cortisol, and testosterone levels were found to be quite low. He was administered stress dose steroids peri-operatively and ultimately discharged on indefinite hydrocortisone therapy and endocrinology follow-up. Our case highlights a serious complication of pituitary adenomas that can occur called pituitary apoplexy which is caused by acute ischemic infarction or hemorrhage in the pituitary. It needs prompt identification and management. Our case also emphasizes the importance of recognizing pituitary apoplexy as one of the causes of sudden onset cranial nerve deficits, as it is a rare presentation of pituitary adenomas that can be taken for a stroke in the Emergency Department.

## Introduction

Pituitary adenomas are the fourth most common intracranial tumors as per the American Association of Neurological Surgeons [1]. Most of them are benign and slow growing. Adenomas larger than 10 mm are called macroadenomas. They are often discovered due to the symptoms they produce by mass effect. Pituitary apoplexy refers to the sudden death or apoptosis of the pituitary gland. It can be caused by an acute ischemic infarction or hemorrhage in the pituitary gland. It can occur when a pituitary tumor outgrows its blood supply. Pituitary apoplexy is a medical and surgical emergency in most cases. Prompt identification and management are vital for better outcomes as also stated by Del Valle et al. [2]. Neurological deficits caused by the mass effect of pituitary tumors and apoplexy can sometimes be misleading toward other diagnoses. We share one such case, which highlights the importance of considering pituitary apoplexy among the differentials of sudden onset cranial nerve deficits.

## Case presentation

A 48-year-old man with a history of cirrhosis, diabetes, hypertension, and end-stage renal disease (ESRD) presented to the Emergency Room for evaluation of a headache, associated with ptosis of the right eye and right-sided blurry vision. Symptoms had started three days prior to presentation and progressively worsened, prompting him to come to the hospital. The headache was sudden in onset, temporal in location, radiating to the frontal region, and progressive in severity. He rated it as 10/10 in intensity at the time of presentation. He did not report any dizziness, numbness, or focal weakness anywhere else in the body. On exam, he was alert and oriented with normal speech. He was mildly hypertensive with a blood pressure of 152/77 mm Hg. The rest of his vitals were within normal range. He was noted to have complete ptosis of the right eye along with medial gaze palsy of the right eye. Pupils were constricted bilaterally with a sluggish response to light. No anisocoria was noted. The fundoscopic examination was limited due to pupillary constriction. Visual fields were noted to be intact to confrontation, and facial movements were symmetric. The rest of his exam was unremarkable. His initial labs were within the expected range of the intradialytic period. CT scan of the head on admission reported no intracranial hemorrhage, infarction, or mass. A CT Angiogram of the head and neck was unremarkable. He was given aspirin 325mg for concern of stroke, and an MRI of the brain was ordered, which was not done until the next day. Meanwhile, he was given metoclopramide, diphenhydramine, and ketorolac for headaches with minimal relief. The findings on the MRI of the brain came as a surprise but explained the patient’s symptoms very well. It revealed a 2.1x2.1x1.4 cm rim-enhancing sellar/suprasellar mass causing a mass effect on the optic chiasm. This was followed by a dedicated pituitary fossa MRI which confirmed enlargement of the pituitary gland consistent with macroadenoma with suprasellar extension and hemorrhage in the lesion, causing optic chiasm compression and displacement upwards. He had developed pituitary apoplexy that likely occurred several days ago. Neurosurgery was consulted immediately, and the patient was scheduled to undergo trans-sphenoidal resection of the tumor; however, unfortunately, surgery had to be delayed for five days as he had received aspirin 325mg on admission. Otolaryngology was consulted for co-management of this procedure and endocrinology was consulted for possible complications after surgery. Hormone levels were checked, and he was found to have low levels of adrenocorticotropic hormone (ACTH) and cortisol, consistent with central adrenal insufficiency, hence, he was started on hydrocortisone replacement therapy. He was also found to have other hormonal abnormalities which are shown in Table 1. Ultimately, he underwent surgery without any complications. He was monitored closely for post-operative diabetes insipidus, however, he did not develop it, partly due to his ESRD. His sodium, glucose, and potassium levels remained within the expected range. Other endocrine labs were monitored as well and he thankfully did not develop any acute drop in his thyroid-stimulating hormone (TSH) or free T4 level. He was given stress dose steroids peri-operatively with subsequent tapering to a maintenance dose. He was finally discharged home on a regimen of hydrocortisone indefinitely and advised to follow up with endocrinology to monitor his TSH, free T4, and testosterone levels to assess his pituitary function and need for replacement therapy. Tumor pathology was consistent with a partially infarcted pituitary adenoma.

**Table 1:** Hormone levels at the time of diagnosis.

Lab	Patient Value	Normal range
Adrenocorticotropic hormone (ACTH)	<9 pg/mL	9-46 pg/nmL
AM cortisol	1.3 mcg/dL	3.7-19.4 mcg/dL
Luteinizing hormone (LH)	0.6 mIU/mL	1.8-8.6 mIU/mL
Follicle-stimulating hormone (FSH)	2.2 mIU/mL	1.5-12.4 mIU/mL
Testosterone	<10 ng/dL	270-1070 ng/dL
Thyroid-stimulating hormone (TSH)	0.74 uIU/mL	0.3-5.0 uIU/mL
Free T4	0.7 ng/dL	0.7-1.7 ng/dL
Prolactin	49 ng/mL	0-19 ng/mL

**Figure 1:**
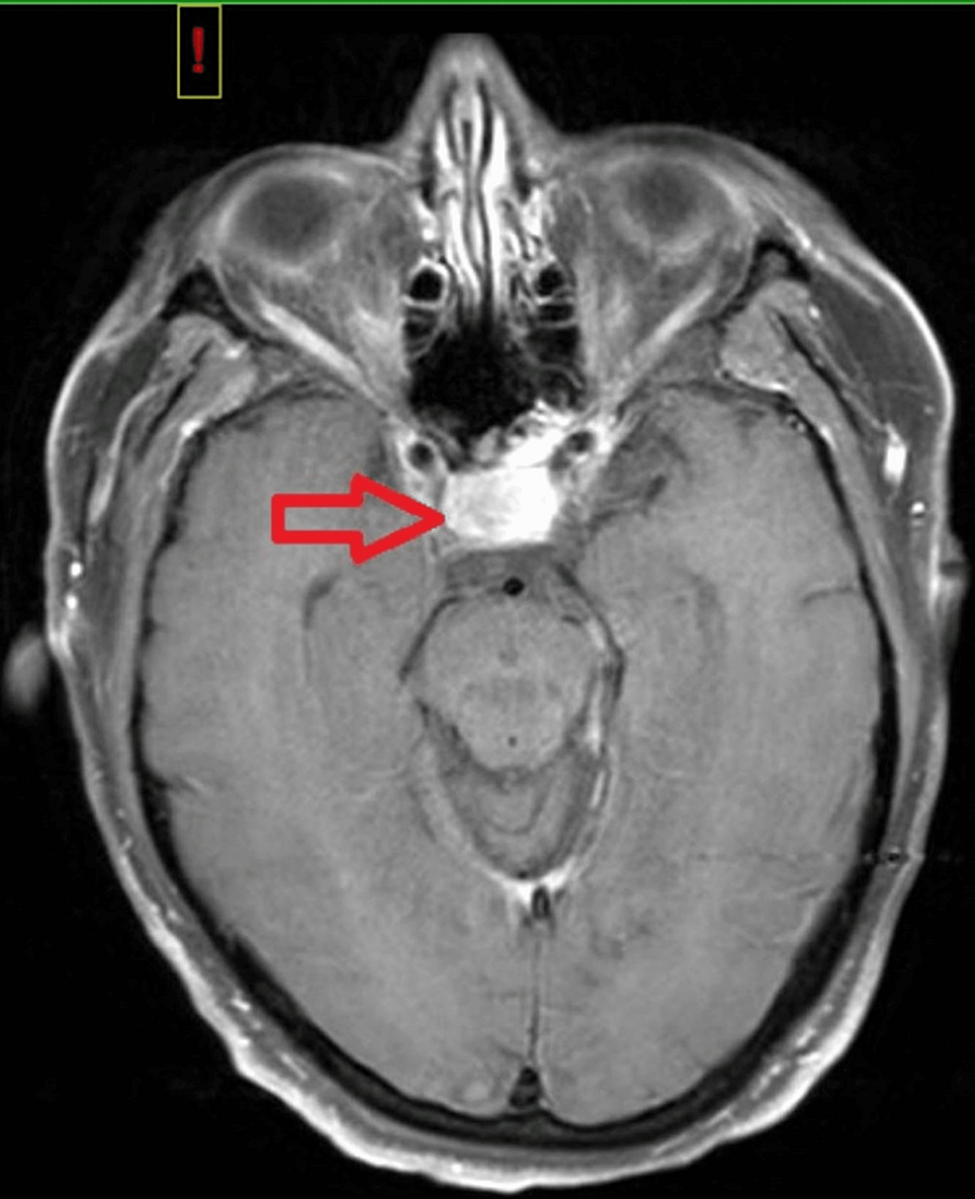
Expanded sella with hemorrhage consistent with macroadenoma with suprasellar extension and elevation of the optic chiasm.

## Discussion

This case highlights the importance of broadening our differentials and including pituitary tumors as a possible etiology when patients present with cranial nerve deficits. It also demonstrates a level of anchoring bias due to which this case was initially diagnosed as a stroke and treated with a loading dose of aspirin, delaying his surgery.

What makes our case more interesting is the fact that the pituitary adenoma was not picked up on the initial CT scan of the head, which is unusual given its size. A subsequent review of the initial CT imaging by a neuroradiologist later stated that although it was present on the initial CT scan but was difficult to see due to streak artifact. Hence, this case also emphasizes keeping in mind the inter-observer variability of interpretations and limitations of imaging modalities. We should not over-rely on imaging reports and should always question them when they don’t correlate clinically.

Pituitary apoplexy is a relatively rare but serious complication of pituitary adenomas which occurs in 2%-12% of the cases as mentioned by Briet et al. [3]. It occurs due to an acute hemorrhage or infarction or even in the pituitary gland. In some cases, a triggering factor may be identified such as elevated intracranial pressure, arterial hypertension, or anticoagulants, to name a few.

The main symptom of pituitary apoplexy is a sudden onset headache which is severe in intensity. Other symptoms may include visual disturbances such as blurry vision, diplopia, visual field cuts, and ptosis. Sani et al. report an unusual case of pituitary apoplexy in the British Journal of Medical Practitioners, 2020 [4] in which a 49-year-old man presented with rather subtle complaints of loss of peripheral vision in one eye and lethargy for two weeks and was otherwise stable. Therefore, it is important to know that despite its severe pathology and consequences, pituitary apoplexy can have varied presentations, and some patients may have only mild symptoms. Hence, it should always be considered in any patient who presents with neuro-ophthalmic deficits, regardless of clinical stability.

CT scan (most of the time) and MRI of the brain are diagnostic as they reveal a pituitary tumor associated with hemorrhage. Prompt diagnosis and management are essential to prevent adverse outcomes, as most of the patients develop pituitary insufficiency as a consequence. Once the diagnosis is made, a multidisciplinary team including a neurosurgeon and an endocrinologist should be consulted to co-ordinate an appropriate management plan as outlined by Rajasekaran et al. [5]. The type and degree of pituitary insufficiency varies between patients. Adrenal insufficiency leading to the adrenal crisis may be life-threatening if left untreated. Our patient developed adrenal insufficiency, however, thankfully, he did not develop an adrenal crisis as he was appropriately started on steroid replacement therapy. Surgery is indicated in patients with worsening neurological symptoms such as ours; however, it remains unclear whether conservative or surgical management has the best outcome for most of the patients.

Central diabetes insipidus (CDI) is a common complication that can occur after pituitary surgery, whereby kidneys excrete an abnormally large volume of dilute urine, resulting in high serum osmolality. Factors that increase the risk of this complication include large tumor size, gross total resection, and intra-operative cerebrospinal fluid leak among others. It is important to recognize and manage CDI in time as it can cause high morbidity and mortality [6]. Post-surgical CDI is mostly transient but can also become permanent. Transient Diabetes Insipidus usually occurs within 24-48 hours after surgery, resolving over the following few days [7]. Our patient was closely monitored for this complication; however, he did not develop CDI most likely due to his underlying renal disease.

## Conclusions

In conclusion, we present the case of a 48-year-old man who presented with headache and unilateral third nerve palsy and was ultimately found to have a pituitary macroadenoma associated with hemorrhage in the pituitary consistent with pituitary apoplexy. He was noted to have compression and displacement of the optic chiasm. He developed adrenal insufficiency as a consequence and was started on hydrocortisone replacement therapy. He ultimately underwent a trans-sphenoidal resection of the pituitary tumor and was discharged with endocrinology and neurosurgery follow-up. Pituitary apoplexy is a serious complication of pituitary adenomas and early diagnosis, and management is essential to improve outcomes.
